# Efficacy of FLU-v, a broad-spectrum influenza vaccine, in a randomized phase IIb human influenza challenge study

**DOI:** 10.1038/s41541-020-0174-9

**Published:** 2020-03-13

**Authors:** Olga Pleguezuelos, Emma James, Ana Fernandez, Victor Lopes, Luz Angela Rosas, Adriana Cervantes-Medina, Jason Cleath, Kristina Edwards, Dana Neitzey, Wenjuan Gu, Sally Hunsberger, Jeffery K. Taubenberger, Gregory Stoloff, Matthew J. Memoli

**Affiliations:** 1 SEEK Central Point, 45 Beech Street, London, EC2Y 8AD UK; 2 h-VIVO Services Ltd, London, UK; 3 Viral Pathogenesis and Evolution Section, Laboratory of Infectious Diseases, Division of Intramural Research, National Institute of Allergy and Infectious Diseases, National Institutes of Health, Bethesda, MD 20892 USA; 4 LID Clinical Studies Unit, Laboratory of Infectious Diseases, Division of Intramural Research, National Institute of Allergy and Infectious Diseases, National Institutes of Health, Bethesda, MD 20892 USA; 5 Biostatistics Research Branch, National Institute of Allergy and Infectious Diseases, Bethesda, MD 20892 USA

**Keywords:** Randomized controlled trials, Translational research

## Abstract

FLU-v, developed by PepTcell (SEEK), is a peptide vaccine aiming to provide a broadly protective cellular immune response against influenza A and B. A randomized, double-blind, placebo-controlled, single-center, phase IIb efficacy and safety trial was conducted. One hundred and fifty-three healthy individuals 18–55 years of age were randomized to receive one or two doses of adjuvanted FLU-v or adjuvanted placebo subcutaneously on days −43 and −22, prior to intranasal challenge on day 0 with the A/California/04/2009/H1N1 human influenza A challenge virus. The primary objective of the study was to identify a reduction in mild to moderate influenza disease (MMID) defined as the presence of viral shedding and clinical influenza symptoms. Single-dose adjuvanted FLU-v recipients (*n* = 40) were significantly less likely to develop MMID after challenge vs placebo (*n* = 42) (32.5% vs 54.8% *p* = 0.035). FLU-v should continue to be evaluated and cellular immunity explored further as a possible important correlate of protection against influenza.

## Introduction

Influenza virus remains at the forefront of public health research due to the high morbidity and mortality associated with yearly epidemics and sporadic pandemics. Seasonal influenza is estimated to cause up to 79,400 deaths in the US and 291,243 to 645,832 deaths globally^1–6^. Influenza vaccination is the primary method available to mitigate the effect of influenza on the world’s population. Current vaccines rely on specific targeting of the major surface protein, hemagglutinin (HA), and are standardized as stimulating anti-HA antibodies as the primary correlate of protection^3^, but these vaccines have limited and somewhat unpredictable efficacy^7^, particularly in those that most require protection such as the elderly, young, and infirm^8^.

Novel correlates of protection are being explored including cellular immunity. Influenza-specific cytotoxic T lymphocytes (CTL) have been shown to be involved in killing and removing cells that are infected with influenza viruses^9–14^. These cells may play an important role in the clearance of influenza virus both before and after clinical symptoms appear. Influenza has been shown to stimulate CTL responses to the internal proteins PB1, PB2, PA, NP, M2, and M1 (refs. ^15–18^), which may stimulate a broadly protective response since these proteins are not as variable as viral surface proteins that typically induce a more specific humoral response.

FLU-v, developed by PepTcell (trading as SEEK), is a peptide vaccine derived from conserved regions of internal proteins aiming to provide a broadly protective immune response against influenza A and B through viral clearance by cytotoxic T cell release of pro-inflammatory cytokines as well as perforin and granzyme. It has demonstrated efficacy in animals^19^ and safety and immunogenicity in Phase I and Phase Ib trials, the latter of which demonstrated cellular immune responses to FLU-v correlated with reduction of viral shedding and reduced symptoms after H3N2 influenza challenge^20,21^.

The objective of this study was to test the efficacy of adjuvanted FLU-v in a healthy volunteer H1N1 challenge model developed by the LID Clinical Studies Unit at the National Institute of Allergy and Infectious Diseases (NIAID)^22^.

## Results

### Demographics and study population

**Fig. 1:**
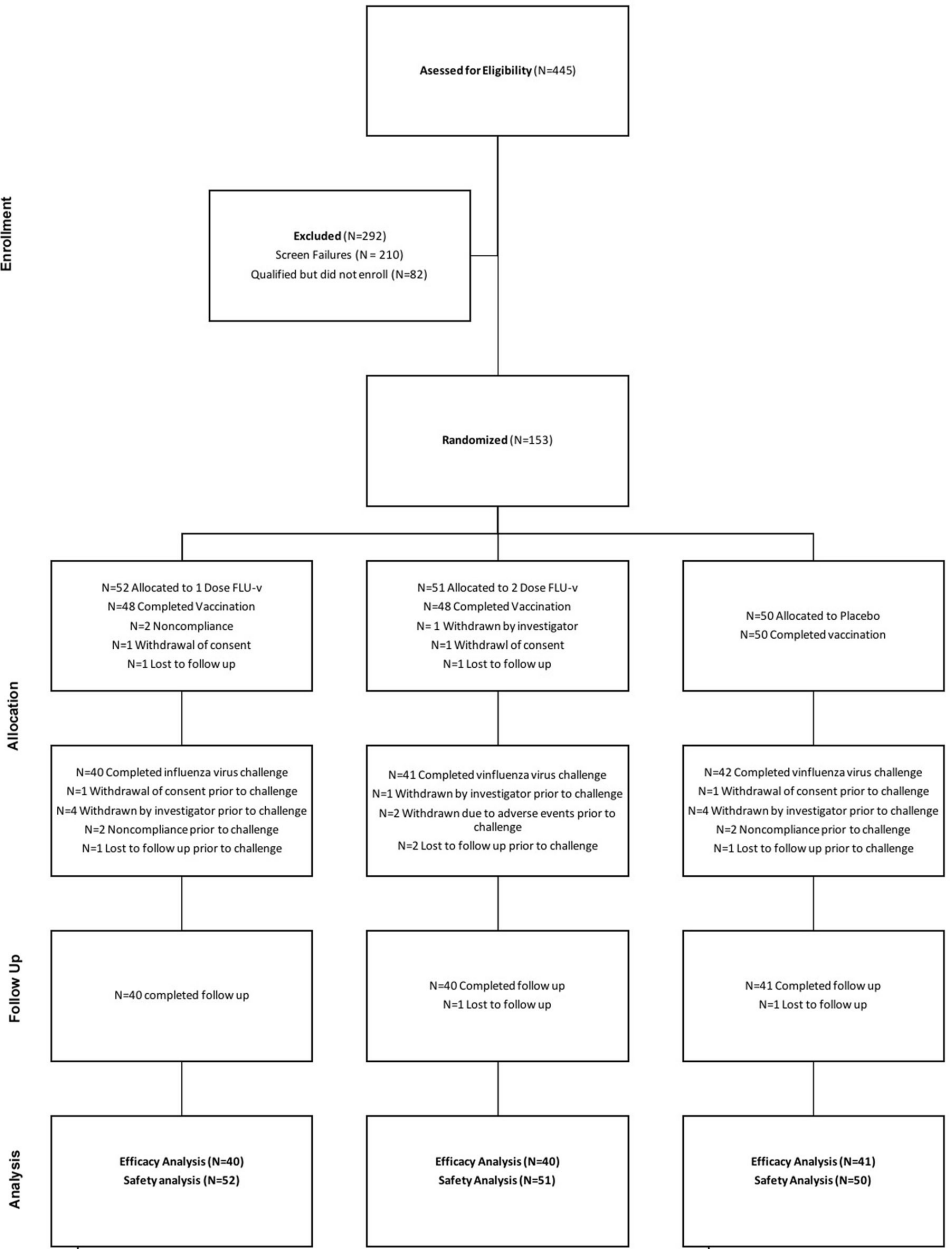
Participant disposition throughout the four phases of the study. The diagram shows the number of subjects (*N*) that moved through various phases of the study as well as when and why people were excluded from the study.

The study activities took place between 18 August 2016 and 31 March 2017. A total of 153 participants were randomized, 52 received one dose of adjuvanted FLU-v followed by one dose of adjuvanted placebo, 51 received two doses of adjuvanted FLU-v, and 50 received two adjuvanted placebo vaccinations. All participants were considered part of the safety population and were evaluated as part of the safety analysis. Only two participants in all of the vaccinated groups were withdrawn prior to challenge due to adverse events (AEs), both of which were considered unrelated to the vaccine. Others were lost to follow-up or withdrew due to lack of further interest in participation, unrelated change in health, or total challenge enrollment completed (Fig. 1). Therefore, of the 153 participants, 123 went on to receive an intranasal challenge with the (H1N1)pdm09 challenge strain. These 123 participants were considered the intent to treat population and were used for the efficacy analysis (Fig. 1). The demographics of each of these populations are summarized in Table 1.

**Table 1:** Study demographics and baseline characteristics.

	1 dose adjuvanted FLU-v (*N* = 40)	2 doses adjuvanted FLU-v (*N* = 41)	Placebo (*N* = 42)	Total (*N* = 123)
Male (%)	28 (70)	30 (73.2)	29 (69.1)	87 (70.7)
Female (%)	12 (30.0)	11 (26.8)	13 (30.9)	36 (29.3)
Mean age (SD)	29.9 (9.3)	27.4 (8.7)	28·8 (7.6)	28.7 (8.6)
White (%)	37 (92.5)	34 (82.9)	33 (78.6)	104 (84.6)
Asian/Asian British (%)	0 (0)	1 (2.4)	1 (2.4)	2 (1.6)
Black/Black British (%)	0 (0)	2 (4.9)	3 (7.1)	5 (4.1)
Chinese (%)	1 (2.5)	0 (0)	0 (0)	1 (0.8)
Hispanic (%)	1 (2.5)	0 (0)	0 (0)	1 (0.8)
Japanese (%)	0 (0)	0 (0)	1 (2.4)	1 (0.8)
Mixed (%)	0 (0)	3 (7.3)	4 (10.0)	7 (5.7)
Other (%)	1 (2.5)	1 (2.4)	0 (0)	2 (1.6)

Overview of age, gender, and ethnicity of all participants enrolled, randomized, and challenged with influenza. This population was used for all primary and secondary analyses.

### Efficacy of FLU-v

The highest rate of mild to moderate influenza disease (MMID) was observed in the placebo group, with 54.8% of participants experiencing MMID. In the two-dose FLU-v group, 36.6% (*p* = 0.075) experienced MMID while 32.5% (*p* = 0.035) experienced MMID in the one-dose FLU-v group, a statistically significant reduction in the primary endpoint vs the placebo group (Table 2).

**Table 2:** Number (percent) of subjects with MMID, symptoms, shedding, or shedding with no symptoms.

	FLU-v 1 × *N* = 40	Placebo *N* = 42	*p* (lower bound, est)	FLU-v 2 × *N* = 41	Placebo *N* = 42	*p* (lower bound, est)
MMID (%)	13 (32.5)	23 (54.8)	*0.035 (1.8, 22.3)	15 (36.6)	23 (54.8)	0.075 (−2.2, 18.2)
No MMID %)	27 (67.5)	19 (45.2)		26 (63.4)	19 (45.2)	
Symptoms (%)	34 (85.0)	37 (88.1)	0.47 (−11.7, 3.1)	30 (73.2)	37 (88.1)	0.074 (−1.7, 14.9)
≥2 symptoms (%)	16 (40.0)	27 (64.3)	*0.024 (3.7, 24.3)	23 (56.1)	27 (64.3)	0.30 (−11.7, 8.2)
Shedding (%)	15 (37.5)	23 (54.8)	0.089 (−3.2, 17.3)	18 (43.9)	23 (54.8)	0.22 (−9.4, 10.9)
Asymptomatic shedding (%)	2 (5.0)	1 (2.4.0)	0.89 (−13.2, −2.6)	5 (12.2)	1 (2.4)	0.99 (−22.1, −9.8)

The incidence of the binary endpoints was calculated and then analyzed using a one-sided Fisher’s exact test with *p* < 0.05 being considered significant (*). The lower boundary for the estimate of the difference (placebo-vaccine) in the percentage of each endpoint is presented that corresponds to a one-sided 95% significance level, along with the estimate of the difference. MMID (mild to moderate disease) was considered positive if a participant had at least one symptom of influenza along with a positive diagnostic test during the quarantine period post influenza challenge.

When evaluating clinical disease, no significant difference in the number of participants experiencing at least one symptom was observed between the groups (Table 2). However, there was a statistically significant reduction in the number of participants who experienced at least two or more symptoms during their infection between those who received single-dose FLU-v and those who received placebo (40% vs 64.3%, *p* = 0.024). A smaller reduction in the two-dose FLU-v group (56.1%) compared to placebo in the participants with at least two or more symptoms (*p* = 0.296) was also observed but not significant (Table 2).

**Fig. 2:**
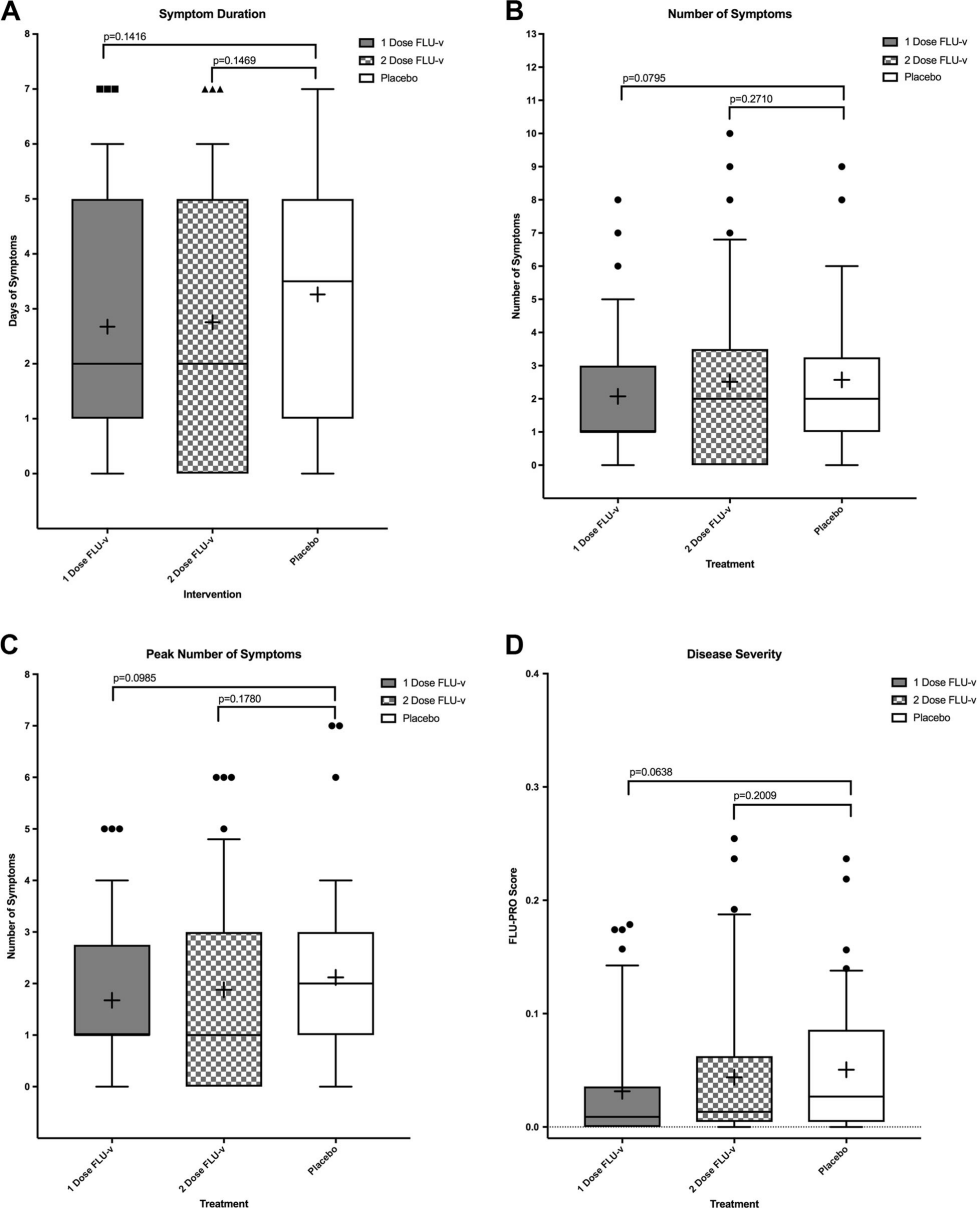
Symptom and disease severity measures were evaluated using a one-sided Wilcoxon rank-sum test at a 0.05 significance level. Box and whisker plots show the median and interquartile range. Error bars span the 10th to 90th percentile. Outliers are indicated as points outside of whiskers. The mean is shown as a “+”. **a** Days of symptoms experienced by participants in each vaccination group after challenge. **b** Number of symptoms experienced by participants in each vaccination group after challenge. **c** Peak number of symptoms in 1 day experienced by participants in each vaccination group after challenge. **d** Total FLU-PRO score (symptom severity questionnaire) for participants in each vaccination group after challenge.

**Fig. 3:**
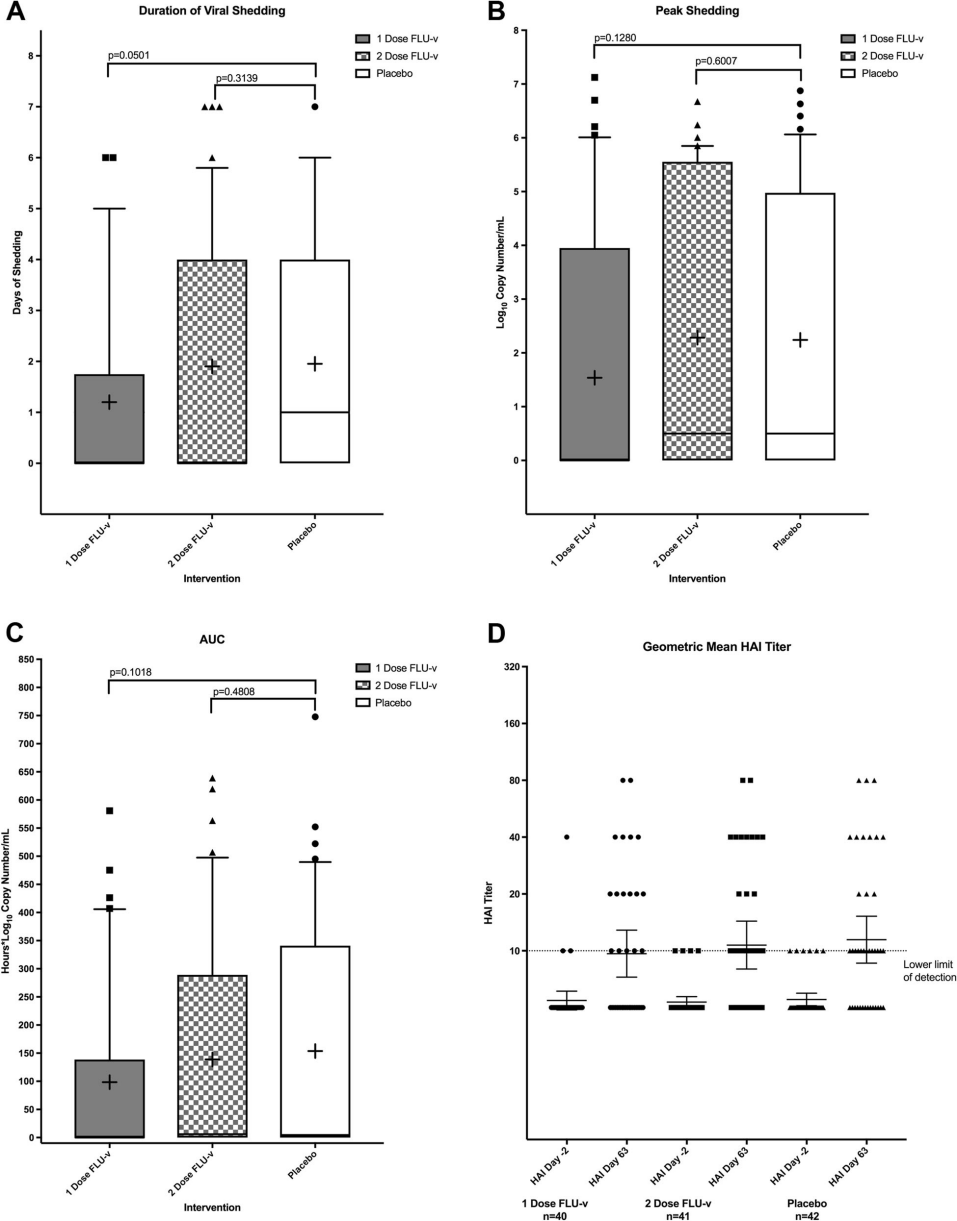
Shedding and AUC was evaluated using a one-sided Wilcoxon rank-sum test at a 0.05 significance level. Box and whisker plots show the median and interquartile range. Error bars span the 10th to 90th percentile. Outliers are indicated as points outside of whiskers. The mean is shown as a “+”. **a** Days of shedding of participants in each vaccination group after challenge. **b** Peak shedding measured by rt-pcr (Log_10_ copies/ml) experienced by participants in each vaccination group after challenge. **c** AUC of participants in each vaccination group after challenge. **d** HAI titers were measured in each of the three groups 2 days before challenge (day −2) and 63 days after challenge). Most participants had undetectable titers prior to challenge. Geometric mean titers are shown here with error bars representing 95% confidence intervals.

Median duration of symptoms and severity of disease as measured by the FLU-PRO questionnaire was reduced in those receiving vaccine compared to the placebo group in both vaccine groups, but the results were not statistically significant (Fig. 2). Non-statistically significant reductions in the mean total number of symptoms and mean peak symptoms were also observed in the one-dose FLU-v group compared to placebo but not for the two-dose FLU-v arm (Fig. 2). Similarly, the presence of shedding was lower in both vaccine groups compared to placebo as was the AUC of total viral shedding, although not statistically significant (Table 2, Fig. 3).

### Safety of FLU-v

Overall, FLU-v vaccination was well tolerated in all participants receiving the vaccine. After vaccination, but before influenza challenge, 98 participants (64%) experienced an AE. The number of participants experiencing one or more AEs in the one-dose adjuvanted FLU-v group was similar to placebo, 55.8% vs 54%, respectively, but a higher percentage of individuals (50% vs 22%) in the one-dose group experienced AEs definitely, probably, or possibly related to vaccination compared to placebo (Table 3). The largest number of individuals experiencing AEs was observed in the two-dose adjuvanted FLU-v group at 82.4%, with 75% of participants experiencing AEs definitely, probably, or possibly, related to vaccination (Table 3). No vaccine related AEs were observed after challenge.

**Table 3:** Summary of adverse events post-vaccination and post-challenge.

	One dose (*N* = 52)	Two doses (*N* = 51)	Placebo (*N* = 50)
Post-vaccination			
Subjects with one or more AE, *n* (%)	29 (55.8)	42 (82.4)	27 (54.0)
AE related to vaccination, *n* (%)			
Definitely related	24 (46.2)	29 (56.9)	7 (14.0)
Probably related	0 (0)	6 (11.8)	1 (2.0)
Possibly related	2 (3.8)	3 (5.9)	3 (6.0)
Unlikely to be related	2 (3.8)	4 (7.8)	8 (16.0)
Not related	10 (19.2)	20 (39.2)	19 (38.0)
AE severity, *n* (%)			
Mild	29 (55.8)	39 (76.5)	25 (50.0)
Moderate	3 (5.8)	8 (15.7)	10 (20.0)
Severe	1 (1.9)	2 (3.9)	0 (0)
Serious AEs, *n* (%)	0 (0)	0 (0)	0 (0)
AEs resulting in withdrawal, *n* (%)	0 (0)	2 (3.9)	1 (2.0)
After challenge			
Subjects with one or more AE, *n* (%)	17 (32.7)	22 (43.1)	27 (54.0)
AE related to vaccine, *n* (%)			
Definitely related	0 (0)	0 (0)	0 (0)
Probably related	0 (0)	0 (0)	0 (0)
Possibly related	0 (0)	0 (0)	0 (0)
Unlikely to be related	2 (3.8)	6 (11.8)	4 (8.0)
Not related	16 (30.8)	19 (37.3)	25 (50.0)
AE severity, *n* (%)			
Mild	16 (30.8)	22 (43.1)	26 (52.0)
Moderate	2 (3.8)	3 (5.9)	4 (8.0)
Severe	0 (0)	1 (2.0)	0 (0)
Serious AEs, *n* (%)	0 (0)	0 (0)	0 (0)
AEs resulting in withdrawal, *n* (%)	0 (0)	0 (0)	0 (0)

Incidence of adverse events occurring before and after influenza challenge are summarized here for each group along with relatedness to vaccine and severity. *N* = number of participants, (%) percentage of total participants in each group.

The most common AE related to vaccination observed in this study was injection site induration (41% of participants). All of these events were mild in intensity except for one participant who experienced injection site induration considered moderate. The highest proportion of participants experiencing this AE were in the two-dose FLU-v group (63%) while fewer were observed in the one-dose FLU-v group (46%) and the placebo group (16%). Injection site pain was observed in 6% of those in the two-dose FLU-v group. No other AEs related to vaccination were observed in greater than 4% of the participants in any of the treatment groups. All of these other AEs were mild in intensity except for two events, one arthralgia and one injection site reaction considered severe. There were no serious AEs identified (Table 3).

### Anti-hemagglutinin antibody titers

Participants were selected who had an HAI titer against the H1N1 challenge strain of <1:40 prior to vaccination with FLU-v. As expected, no significant rise in hemagglutinin inhibition (HAI) titer was observed after vaccination and all but one participant had an HAI titer of <1:40 after vaccination on the day prior to H1N1 challenge. Participants in all three groups demonstrated a statistically significant rise in geometric mean HAI titer after viral challenge from their pre-challenge baseline (Fig. 3d).

## Discussion

The results of this study demonstrate that a peptide-based vaccine designed to induce influenza-specific T cell immunity (in the absence of inducing antibody responses against HA) can provide some protection against influenza. At least one other peptide-based universal influenza vaccine candidate inducing T cell immunity has completed phase I and II trials, but that vaccine has not demonstrated efficacy via a primary outcome^23,24^. However, the ability of peptide vaccines to alter the functionality of T cells has been previously observed^25^, and data from various studies demonstrate that low doses induce high T cell avidity while high peptide concentrations favor low avidity T cells and inhibition of the T cell proliferative response^26^. This can affect the available T cell repertoire in vivo and subsequent pathogen clearance. A high and low zone tolerance after immunization with different doses of antigen exists and must be empirically determined^27^. The optimal dose and formulation of FLU-v was identified in preclinical and clinical studies^20,21^. The dose administered in this trial, 500 ug, provided the best T cell-driven immune responses that correlated with reduction in virus shedding and symptoms in a small Phase IB H3N2 influenza human challenge^21^.

In this study single-dose adjuvanted FLU-v was safe and efficacious in that it induced a statistically significant reduction in the number of participants positive for MMID compared to placebo. These data demonstrate the efficacy of this broad-spectrum, “universal” vaccine candidate and do so in a healthy volunteer human challenge model. In addition, every other secondary endpoint measured to evaluate disease severity and viral shedding, although not statistically significant, demonstrated reductions in favor of single-dose FLU-v.

Previous human studies were carried out administering a single-dose of FLU-v, but in this study two doses were tested in humans. Although the two-dose regimen showed a reduction in MMID compared to placebo, this was not statistically significant. The study was only powered to compare the active groups to placebo so there was no comparison of the active groups. Larger studies are needed to directly compare the single to two doses of adjuvanted FLU-v. However, the lack of a statistically significant effect of two doses of adjuvanted FLU-v may be explained by recent data in mice demonstrating that T cells from thrice vaccinated mice were significantly less effective in adoptive transfer studies than T cells from mice receiving a single vaccination^28^. In that study the researchers observed an increase in the number of regulatory T cells in animals that had received multiple vaccinations. Elimination of these regulatory cells during the second and third vaccinations resulted in a recovery of therapeutic efficacy. In addition, previous studies have demonstrated that repeated exposure within a short time or prolonged continuous exposure to antigen stimulation can result in T cell exhaustion, characterized by a higher level of activation and differentiation of T cells than seen in acute stimulation leading to reduced effector function. This can include a reduction in the number of cells able to produce cytokines leading to a failure to provide help to other lymphocytes to stimulate an effective antigen response^29,30^. Further study of peripheral blood mononuclear cells and whole-blood RNA collected after vaccination with FLU-v need further evaluation to test the above hypotheses and to define the protective immune responses being generated.

The major limitation of this study is the generalizability of the human challenge model. The human challenge participants in this study represent the healthiest of individuals who could acquire an influenza infection, are somewhat homogeneous, and the disease demonstrated by these participants was therefore mild overall. Less than 10% of participants in a typical human challenge study would likely be medically attended, and even then, most if not all would be considered mild cases of influenza. Therefore, it is possible that conclusions observed in such a model may not be comparable to a general population with a broader spectrum of illness. However, this narrow spectrum of illness also makes it more difficult to detect efficacy of a vaccine suggesting that a vaccine like FLU-v that demonstrates efficacy in this setting could have a more amplified effect in a real-world setting where the spectrum of disease severity is much broader. In addition, further exploration of FLU-v in the elderly may be very useful as the ability to generate antibody responses against the antigens in seasonal influenza vaccine decreases with age leading to lower efficacy of seasonal vaccination while Th1 cellular responses remain unaffected^31^.

Single-dose adjuvanted FLU-v demonstrated a statistically significant reduction in the number of individuals who developed at least two symptoms of influenza, regardless of shedding. FLU-v was able to offer protection that reduced the severity of illness, even in this very healthy population. In addition to this, consistent non-statistically significant reductions of duration of symptoms, total number of symptoms, peak number of symptoms, symptom severity, incidence of shedding, viral shedding AUC, peak shedding, and shedding duration further strengthen this conclusion. The primary endpoint of MMID is a strict definition of influenza infection and in a typical human population exposed to influenza naturally a broader spectrum of illness would occur, therefore making it likely that the effect of this vaccine on disease severity may be even greater than observed here.

FLU-v does not contain HA antigens and as such does not provide protection by inducing neutralizing antibodies against HA. As expected, FLU-v did not significantly increase HAI titers, as most participants were observed to have low HAI titers after vaccination at the time of influenza challenge. Overall geometric mean HAI titers did increase after infection indicating exposure to the challenge virus. Titers remained low as has been observed before in human challenge models that screen participants for low HAI titers prior to challenge^32–34^, suggesting that screening for low HAI titer in challenge trials may select for a higher proportion of individuals than the 10–20% found in the general population that do not mount good antibody responses against the HA of influenza^35–37^. It is possible that these individuals may have a stronger cellular response to influenza than those who have a strong anti-HA head humoral response. This in turn could have increased the efficacy of FLU-v in this study; however, there is no evidence to suggest that protective cellular responses such as those potentially induced by FLU-v are downregulated by strong systemic antibody responses.

The results of this study were consistent with what was observed in previous phase I trials and a recently conducted phase IIb trial in the field carried out in the Netherlands^38^. In this 175-participant study, one dose of adjuvanted FLU-v was shown to be safe and to induce long-lasting cell-mediated immune responses.

This study demonstrated that the T cell strategy employed by FLU-v has potential to offer protection against influenza infection as a vaccine. These results demonstrate that T cell immunity against conserved regions of the influenza virus is likely to be an important component for “universal” vaccine strategies. Larger studies with FLU-v can further describe the cellular immune response and evaluate how the vaccine interacts with influenza disease in different cohorts, allowing for informed clinical development of FLU-v and other vaccine strategies that induce T cell immunity.

The need for better, more broadly protective vaccines against influenza is a high priority worldwide, and few new vaccines have demonstrated efficacy in humans. The efficacy of FLU-v in this wild-type human influenza challenge study along with the supporting data from previous trials in the field should be further examined in larger field trials where efficacy of FLU-v will be evaluated against a broader set of influenza strains and wider spectrum of disease to determine if these results will be broadly applicable.

## Methods

### Clinical trial

A randomized, double-blind, placebo-controlled, single-center, phase IIb efficacy and safety trial was conducted at h-VIVO Services Ltd (London, UK). The study (ClinicalTrials.gov: NCT03180801, EudraCT: 2016-002134-74) was approved by UK’s Medicines and Healthcare products Regulatory Agency and the North East-York Research Ethics Committee and registered on 8 June 2017. All participants signed informed consent, and the study was conducted in accordance with the provisions of the Declaration of Helsinki and Good Clinical Practice guidelines.

Participants were recruited in August of 2016. Eligible volunteers were healthy, between the ages of 18 and 55, and had pre-challenge HAI titers to the challenge strain of <1:40 within 90 days prior to entering the study site. Participants were subjected to a complete detailed physical examination as well as obtaining an ECG and spirometry. Participants were excluded if any medical issue was found that placed them at risk of complication from vaccination or influenza challenge.

Adjuvanted FLU-v or placebo was administered subcutaneously on days −43 and −22, prior to intranasal challenge on day 0 with the wild-type (H1N1)pdm09 virus. All participants received two vaccinations 21 days apart. Participants were randomized 1:1:1 into three groups on day −43, the first day of vaccination, based on randomization codes generated by the NIAID statistician in R. The participants, investigators, and all other clinical and non-clinical staff remained blinded to this allocation until after database lock and unblinding. The placebo group received two doses of the adjuvanted placebo, the one-dose FLU-v group received adjuvanted FLU-v and then adjuvanted placebo, and the two-dose FLU-v group received two doses of adjuvanted FLU-v. All groups proceeded to intranasal viral challenge.

A total of 10^7^ TCID_50_ of influenza challenge virus was delivered intranasally as previously described^22^. All participants were required to remain in isolation for approximately 10 days. Participants were discharged on day 7 post-challenge if they were afebrile, were clinically and hemodynamically stable, and had two negative diagnostic tests for influenza on consecutive days. Participants attended follow-up visits on days 35 and 63.

The primary endpoint of the study was to determine the effect of FLU-v on reducing the incidence of MMID, defined as detectable viral shedding by an FDA approved clinical testing method for influenza plus at least one clinical symptom/sign of influenza at any time during the inpatient portion of the study. Secondary outcome measures included incidence of viral shedding, incidence of influenza symptoms, incidence of two or more influenza symptoms, total quantity of symptoms, quantity of viral shedding, symptom severity, and safety during the inpatient portion of the study.

### Clinical evaluation

Clinical symptoms were assessed using the FLU-PRO Symptom Severity Assessment Tool, a participant-directed questionnaire designed to assess influenza disease severity that has been validated in both natural infection and influenza challenge studies^39–41^. In addition, a physician assessment was also performed daily for 10 days to determine the presence or absence of influenza signs/symptoms. Those assessed included arthralgia, chills, conjunctivitis, coryza, diarrhea, dry cough, dyspnea/shortness of breath, fatigue/tiredness, fever (>38.0 °C), headache, myalgia, nausea, productive cough, rhinorrhea, sore throat, and sweats, as well as oxygen saturation decrease by ≥3% from baseline. The duration of symptoms, total number of symptoms experienced, and peak number of symptoms were calculated based on this physician’s assessment.

### Safety evaluation

AEs were captured from the time informed consent was signed until the subject’s last study follow-up visit and were coded using MedDRA system organ class and preferred term. Solicited local and systemic signs of reactogenicity were recorded on AE diary cards issued to all study participants and filled out for 21 days after each vaccination. Participants were followed for a total of 63 days post influenza challenge to identify vaccine-related AEs.

### Viral detection and quantitation

Nasopharyngeal swab samples were collected to evaluate viral shedding. MMID and shedding duration was based on viral detection performed using the Luminex NxTAG Respiratory Pathogen Panel assay (Luminex, Austin, Tx). Quantitation was performed using a reverse transcription quantitative polymerase chain reaction (RT-qPCR) assay targeting the matrix segment of the challenge virus using standard methods. The assay standard was obtained from an RNA produced by in vitro transcription from a plasmid with a sequence specific to the challenge virus. The lower limit of quantitation was 2.30 log_10_ copies/ml (200 copies/ml). An arbitrary value of 1.30 log_10_ copies/ml (20 copies/ml) was given to samples returning a detected but unquantifiable result. Viral shedding peak and viral shedding quantitation (area under the curve) were calculated from day 1 to day 7 post-inoculation.

### Vaccine

FLU-v is a lyophilized vaccine composed of four short peptides; FLU-5 (32aa), FLU-7 (21aa), FLU-8N (20aa), and FLU-10 (24aa)^19,20^. These peptides originate from conserved regions in internal proteins M1, NPA, NPB, and M2 respectively. The peptides are manufactured in the solid phase by fluorenylmethoxycarbonyl (Fmoc) chemistry, reconstituted, filtered, and lyophilized. It was administered by subcutaneous injection as a water-in-oil emulsion (50:50) with water for injection and Montanide ISA-51 adjuvant (Seppic, France) with 500 μg FLU-v in a final volume of 0.5 ml. The placebo was prepared by emulsifying 0.25 ml of water for injection and 0.25 ml of Montanide ISA-51. Montanide ISA-51 is a ready to use adjuvant composed of light mineral oil and a surfactant (mannide monooleate from vegetable oil) designed to form water-in-oil emulsions in order to enhance the immune response against targeted antigens.

### Challenge virus

A cell-grown, reverse genetics produced wild-type (H1N1)pdm09 influenza A virus was used for intranasal challenge. This GMP-manufactured challenge virus was developed in NIAID by the LID Clinical Studies Unit and has been described in previous trials^22,33^. A total of 10^7^ TCID_50_ of challenge virus in sterile saline was delivered in two 500 μl preferred term doses intranasally using the MAD^TM^ Nasal sprayer device (Wolfe-Tory Medical, Inc., Salt Lake City, UT).

### Statistical analysis

The MMID endpoint was used to previously validate this challenge model^22^; therefore, this study was designed with 80% power to detect a reduction in MMID from a predicted placebo rate of 70% MMID to 40% expected in the treatment arms. A one-sided analysis at the 0.05 level of significance was pre-specified. A one-sided test was specified since this is a phase II study and it is only of interest to show activity of the vaccine groups over placebo, it was not of interest to show activity of placebo over vaccine. This decision allowed a smaller sample size to obtain adequate power. Statistical significance of incidence of MMID as well as other binary endpoints was tested using a one-sided Fisher’s exact test with *p* < 0.05 being considered significant as per the approved pre-study statistical analysis plan. Lower 95% boundaries of the difference in proportions were calculated using the method of Fay et al.^42^ that correspond to the Fisher’s exact test. Continuous disease severity measures were compared using a one-sided Wilcoxon rank-sum test with *p* < 0.05 considered significant. HAI titers were evaluated by calculating geometric means and forming two-tailed 95% confidence intervals. Non-overlapping confidence intervals were considered significant.

### Reporting summary

Further information on research design is available in the Nature Research Reporting Summary linked to this article.

## Supplementary information

Reporting summary

## Data Availability

The authors declare that all data from this study are available from the authors, in this report, and through ClinicalTrials.gov #NCT03180801.
